# Supplementary material for: Nonlocal electrical detection of reciprocal orbital Edelstein effect
Source: Nat Commun. 2025 Jul 10;16:6380. doi: 10.1038/s41467-025-61602-7 (PMC12246179; doi:10.1038/s41467-025-61602-7)
Supplement: Supplementary file 1 — Supplementary Information [file 41467_2025_61602_MOESM1_ESM.pdf]

Supplementary Information

Nonlocal Electrical Detection of Reciprocal Orbital Edelstein Effect

Weiguang Gao[1,†], Liyang Liao[1,†], Hironari Isshiki[1], Nico Budai[1], Junyeon Kim[2,3], Hyun-Woo Lee[4,5], Kyung-Jin Lee[6], Dongwook Go[7,8], Yuriy Mokrousov[7,8], Shinji Miwa[1,9], and Yoshichika Otani[1,2,9,*]

[1] *Institute for Solid State Physics, The University of Tokyo, Kashiwa, Chiba 277-8581, Japan*

[2] *Center for Emergent Matter Science, RIKEN, Wako, Saitama 351-0198, Japan*

[3] *National Institute of Advanced Industrial Science and Technology (AIST), Research Institute for Hybrid Functional Integration, Tsukuba, Ibaraki 305-8568, Japan*

[4] *Department of Physics, Pohang University of Science and Technology, Pohang 37673, Korea.*

[5] *Asia Pacific Center for Theoretical Physics, Pohang 37673, Korea.*

[6] *Department of Physics, Korea Advanced Institute of Science and Technology, Daejeon 34141, Korea*

[7] *Institute of Physics, Johannes Gutenberg University Mainz, Mainz 55099, Germany.*

[8] *Peter Grünberg Institut, Forschungszentrum Jülich, Jülich 52428, Germany.*

[9] *Trans-scale Quantum Science Institute, The University of Tokyo, Bunkyo-ku, Tokyo 113-0033, Japan*

[†] These authors contributed equally.

[*] e-mail: yotani@issp.u-tokyo.ac.jp

Table of contents

Supplementary information 1

Nonlocal electrical detection of reciprocal orbital edelstein effect 1

Table of contents 2

Section 1. The distribution of oxygen element in $Al_2O_3/CuO_x/Cu$ nanowire nanowire 3

Section 2. The anisotropic magnetoresistance of ferromagnets 4

Section 3. COMSOL simulation for Hall voltage induced by stray field and bypass current 5

Section 4. Orbital accumulation measured in local transport structure 14

Section 5. The role of spin current injected by electric current 18

Section 6. The nonlocal orbital distribution model which considers bypass current 20

Section 7. The analysis of the angle dependence 24

Section 8. Cu thickness dependence measured in local transport structure 27

Section 9. Temperature dependence measured in local transport structure 29

Section 10. Control experiment with Au/Cu nanowire 34

Section 11. The resistivity of Cu and $Co_{25}Fe_{75}$ and the temperature dependence of bypass current 36

Section 12. Temperature-dependent multiple-step hopping 39

Reference 41

Section 1. The distribution of oxygen element in $Al_2O_3/CuO_x/Cu$ nanowire

Due to the fabrication process, the Cu layer in our sample was exposed to air for 10 minutes before Al_2O_3 deposition, likely resulting in a thin oxidized Cu (CuO_x) layer between Al_2O_3 and Cu. Scanning transmission electron microscopy (STEM) and energy-dispersive X-ray spectrometry (EDX) were used to characterize oxygen distribution (Fig. S1a). Fig. S1b presents the EDX line near the Al_2O_3/Cu interfaces. According to the analysis, oxygen distributes with a concentration gradient confined within ~3 nm below the Cu surface, consistent with previously reported observations of Cu surface oxidation[1–3].

Fig. S1

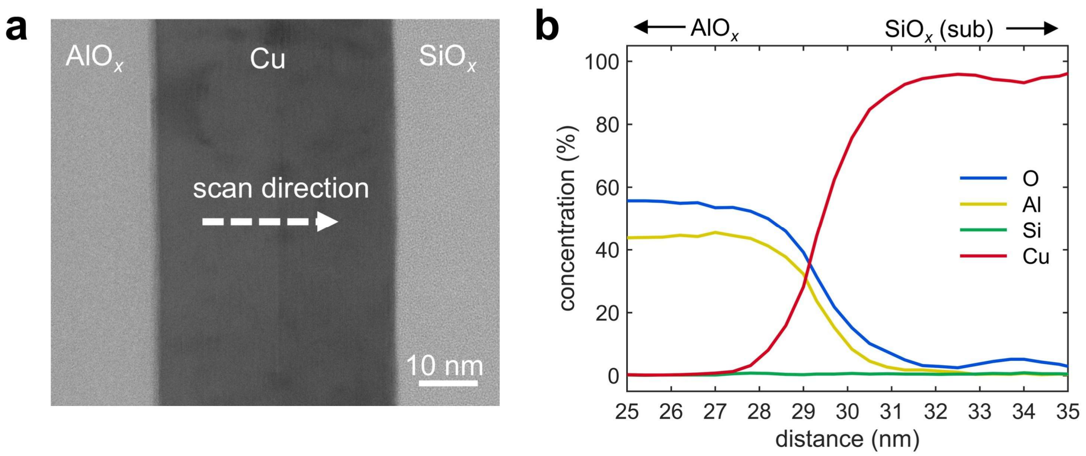

Fig. S1 | STEM and EDX analysis results. a, STEM image for $Al_2O_3/CuO_x/Cu$ sample, representing the observed area for the EDX analysis. **b,** O, Al, Si, Cu atom EDX line profile near the Al_2O_3/Cu interface. The sample was deposited on SiO_2/Si substrate.

Section 2. The anisotropic magnetoresistance of ferromagnets

The anisotropic magnetoresistances (AMR) were measured to estimate the saturation field of magnetization by using the four-probe measurements method and applying an external magnetic field along the hard axis of ferromagnets (FMs). The AMR signals of $Co_{25}Fe_{75}$, $Co_{50}Fe_{50}$, and $Ni_{81}Fe_{19}$ (20 nm thick and 100 nm wide) are shown in Fig. S2a, Fig. S2b, and Fig. S2c, respectively. In nonlocal transport measurements, the amplitude of external magnetic fields is set to 1.25T, which is large enough to saturate the magnetizations.

Fig. S2

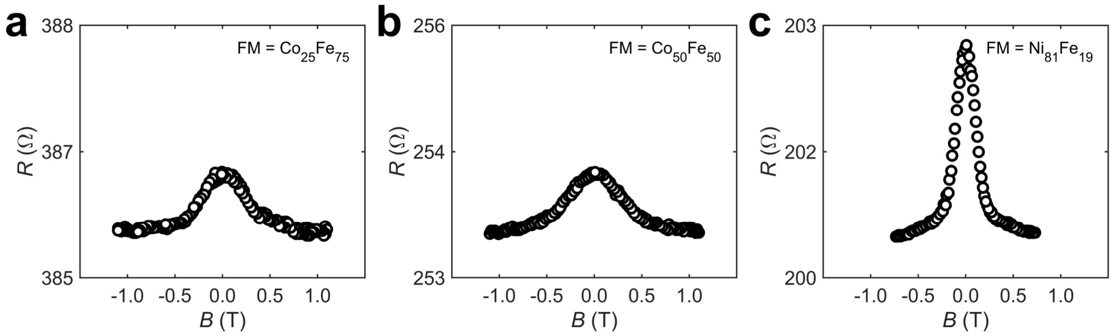

Fig. S2 | Measurement of anisotropic magnetoresistance (AMR) of different FMs. a, b, c, The typical AMR result of $Co_{25}Fe_{75}$ (**a**), $Co_{50}Fe_{50}$ (**b**) and $Ni_{81}Fe_{19}$ (**c**). Here, t_{FM} = 20 nm and w_{FM} = 100 nm.

Section 3. COMSOL simulation for Hall voltage induced by stray field and bypass current

The bypass current in nonlocal measurement

Consider our direct orbital Edelstein effect (DOEE) measurement (direct measurement) structure (see Fig. 1a in main text). When a charge current density j_c flows in the longitudinal $Al_2O_3/CuO_x/Cu$ nanowire, which lies along the y-axis (denoted as Cu_y nanowire hereafter), it shunts to the transverse $Al_2O_3/CuO_x/Cu$ nanowire which lies on x-axis (denoted as Cu_x nanowire hereafter), as shown in Fig. S3a. The magnitude of bypass charge current density j_{by} can be sizeable when the separation distance d between generator and detector is comparable to the width of Cu_x nanowire W[4,5] and induce a significant error in our evaluation of the lateral decay length of orbital accumulation λ_o.

The bypass current can induce both y-polarized and x-polarized orbital angular momenta (OAM), as shown in Fig. S3b. Although the y-polarized orbital accumulation can be generated by the x-component of bypass current density j_{by}^{x} through DOEE, the positive (induced by j_{by}^{+x}) and negative polarized component (induced by j_{by}^{-x}) neutralize each other, leaving no net impact. Therefore, only the y-component of bypass current density j_{by}^{y} give rise to the net contribution to the detected signals in our experiments, as shown in Fig. S3c. The detail discussion on bypass effect is in Section 6.

Fig. S3

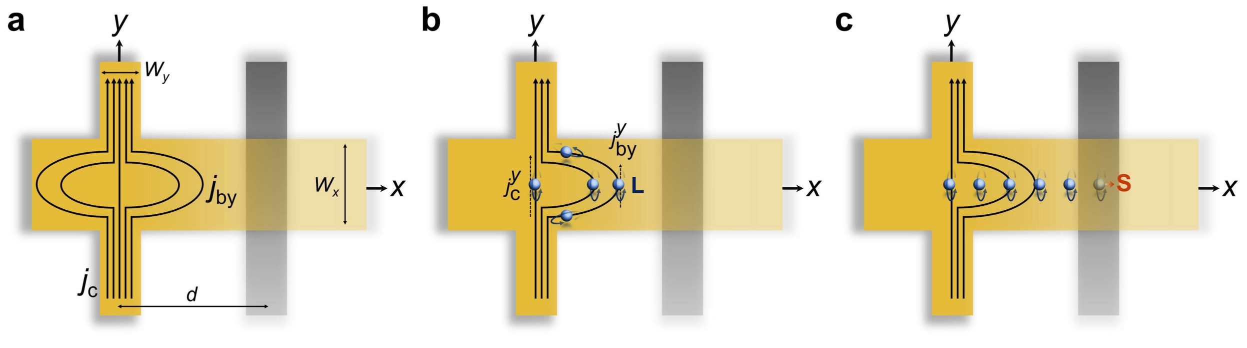

Fig. S3 | The bypass effect of electric current in nonlocal measurement. a, The systematic illustration for the combined contribution of bypass effect of charge current and the lateral distribution of OAM (**L**). The charge current applied to the Cu_y nanowire shuts to the Cu_x nanowire. **b,** Subsequently, the bypass current density induces nonequilibrium OAM at location x (middle panel). **c,** Lastly, all orbital accumulation undergoes a decay before converting to SAM (**S**) and inducing a nonlocal orbital response in FM (right panel). Thus, the signal (nonlocal OEE resistance) is proportional to the volume integration of the final orbital accumulation density by considering the above three stages.

The stray field in nonlocal measurements

Given the short channel length between Cu_y and FM nanowires, in the neighbor where stray field $\mathbf{B}_{\text{stray}}$ and bypass current density $\mathbf{j}_{\text{by}}$ orthogonally coexist, a Hall electric field can be induced. We conducted an analytical discussion and COMSOL simulation to study the artifact induced by the Hall voltage. Given that the length of FM nanowires is much longer than their width and thickness, we assume that the stray field has no y-component and is homogenous along the y-direction in the analytical discussion. Since both $\mathbf{B}_{\text{stray}}$ and $\mathbf{j}_{\text{by}}$ are inhomogeneous, the Hall effect contribution is rather complicated. We hence focus on the leading order contribution from the Hall effect, whose existence relies on the homogeneous parts of the $\mathbf{B}_{\text{stray}}$ and $\mathbf{j}_{\text{by}}$ within a cuboid of the Cu nanowire and ignore the higher order contribution that relying on the inhomogeneity of the $\mathbf{B}_{\text{stray}}$ and $\mathbf{j}_{\text{by}}$ within a cuboid of the Cu nanowire.

Analytical discussion of Hall effect induced by stray field and bypass current

Considering the direct measurement, the detected signal V reflects the potential difference between FM terminal and the right arm of Cu_x nanowire (Fig. S4a), which include the vertical potential difference (V_z) at the Cu/FM junction and x-direction potential (V_x) difference between the Cu/FM junction and right end of Cu_y nanowire. Thus, only in the region B where

$\mathbf{B}_{\text{stray}}$ and $\mathbf{j}_{\text{by}}$ satisfies $E_z \sim -j_{\text{by}}^{y} B_{\text{stray}}^{x}$ (Fig. S4b) and in the region A where $\mathbf{B}_{\text{stray}}$ and $\mathbf{j}_{\text{by}}$ satisfies $E_x \sim j_{\text{by}}^{y} B_{\text{stray}}^{z}$ (Fig. S4c), the Hall effect contributes to the detected signal *V*.

Considering the inverse measurement, the measured signal *V* reflects the *y*-direction potential difference (V_y) between two ends of Cu_y nanowires (Fig. S5a). Thus, only in the region B where $\mathbf{B}_{\text{stray}}$ and $\mathbf{j}_{\text{c}}$ satisfies $E_y \sim j_{\text{c}}^{z} B_{\text{stray}}^{x}$ (Fig. S5b) and in the region A where $\mathbf{B}_{\text{stray}}$ and $\mathbf{j}_{\text{c}}$ satisfies $E_y \sim -j_{\text{c}}^{x} B_{\text{stray}}^{z}$ (Fig. S5c), the Hall effect contributes to the detected signal *V*.

We briefly analyze the region C, D and E, where stray field and bypass current density orthogonally coexist but no contribution is expected. In the direct measurement, region C, D and E allow $E_x \sim j_{\text{c}}^{y} B_{\text{stray}}^{z}$, but the measurement circuit along the FM terminal and the right arm of Cu_x nanowire cannot pick up this electric field. In the inverse measurement, region C, D and E have $E_y \sim j_{\text{c}}^{x} B_{\text{stray}}^{z}$, but given that B_{stray}^{z} is homogeneous along the *y*-direction and the net current I_x is zero, the net contribution of this electric field component to the measured signal is vanishing. Note that the reciprocity law requires a region to contribute to the signal in both of the measurements, or neither of the measurements, so that the A and B regions contribute to both the direct and inverse measurements and C, D, E have vanishing contribution (at the leading order) to both of them. We summarized the Hall effect produced by $\mathbf{B}_{\text{stray}}$ and $\mathbf{j}_{\text{by}}$ and their contribution to the measured signal in table 1 (direct measurement) and table 2 (inverse measurement) below.

Table 1 | Hall effect in direct measurement.

Direct	B^x_{stray}	B^y_{stray}	B^z_{stray}
j^x_{by}	● Non-orthogonal.	● $B^y_{\text{stray}} = 0$	● $E_y \sim -j^x_{\text{by}} B^z_{\text{stray}}$<br>● Not detected by direct measurement.
j^y_{by}	● $E_z \sim -j^y_{\text{by}} B^x_{\text{stray}}$	● Non-orthogonal.<br>● $B^y_{\text{stray}} = 0$	✓ $E_x \sim j^y_{\text{by}} B^z_{stray}$
j^z_{c}	● j^z_{c} does not exist.	● j^z_{c} does not exist.<br>● $B^y_{\text{stray}} = 0$	● Non-orthogonal.<br>● j^z_{c} does not exist.
Region	Hall effect		
A	$E_x \sim j^y_{\text{by}} B^z_{\text{stray}}$.		
B	$E_z \sim -j^y_{\text{by}} B^x_{\text{stray}}$.		
C	$E_x \sim j^y_{\text{c}} B^z_{\text{stray}}$. Yet the E_x is not in the measurement circuit.		
D	$E_x \sim j^y_{\text{c}} B^z_{\text{stray}}$. Yet the E_x is not in the measurement circuit.		
E	$E_x \sim j^y_{\text{c}} B^z_{\text{stray}}$. Yet the E_x is not in the measurement circuit.		

Table 2 | Hall effect in inverse measurement.

Inverse	B^x_{stray}	B^y_{stray}	B^z_{stray}
j^x_{by}	● Non-orthogonal.	● $B^y_{\text{stray}} = 0$	✓ $E_y \sim -j^x_{\text{by}} B^z_{\text{stray}}$
j^y_{by}	● $E_z \sim -j^y_{\text{by}} B^x_{\text{stray}}$<br>● Not detected by inverse measurement.	● Non-orthogonal.<br>● $B^y_{\text{stray}} = 0$	● $E_x \sim j^y_{\text{by}} B^z_{stray}$<br>● Not detected by inverse measurement.
j^z_{c}	✓ $E_y \sim j^z_{\text{c}} B^x_{\text{stray}}$	● $B^y_{\text{stray}} = 0$	● Non-orthogonal.
Region	Hall effect		
A	$E_y \sim -j^x_{\text{by}} B^z_{\text{stray}}$.		
B	$E_y \sim j^z_{\text{c}} B^x_{\text{stray}}$.		
C	$E_y \sim -j^x_{\text{by}} B^z_{\text{stray}}$. Yet no leading order contribution because $\int j^x_{\text{by}} dydz = 0$.		
D	$E_y \sim -j^x_{\text{by}} B^z_{\text{stray}}$. Yet no leading order contribution because $\int j^x_{\text{by}} dydz = 0$.		
E	$E_y \sim -j^x_{\text{by}} B^z_{\text{stray}}$. Yet no leading order contribution because $\int j^x_{\text{by}} dydz = 0$.		

COMSOL simulation

Having established the symmetric analysis of the Hall effect in each part of the devices, we hereby study the Hall voltage induced by stray field and bypass current in both direct and inverse measurements through COMSOL simulations. We first estimated the Hall voltage in the direct measurement using the simulated stray field and bypass current density at two specific point A and B, corresponding to the A and B regions in the previous section. Point A is located 75 nm to the right of the FM nanowire, while point B is directly above the FM nanowire. Both points are positioned along the center axis of the Cu_x nanowire.

At point A, the z component of the stray field is 0.083 T, and the bypass current density is $2.3 \times 10^9\ \mathrm{A/m^2}$, resulting in a Hall electric field $0.01\ \mathrm{A/m}$ along the x direction (taking a Hall coefficient[6] of Cu $R_\mathrm{H} = 5.3 \times 10^{-11}\mathrm{m^3A^{-1}s^{-1}}$). Considering a characteristic distance of 100 nm in the nanowire device, the Hall voltage is 1 nV. At point B, the x-component of the stray field is -0.14 T and the bypass current density is $1.0 \times 10^{10}\ \mathrm{A/m^2}$, resulting in a Hall electric field $0.074\ \mathrm{A/m}$ along the z direction. Using the thickness 40 nm of the Cu nanowire, the Hall voltage is 3 nV. Hence, the Hall effect contribution is at the order of 1nV, which is approximately two orders of magnitude smaller than the experimentally measured signal (100 nV).

To achieve a more precise estimation of the Hall voltage, we refined our approach by dividing the original nonlocal structure into n hypothetical finite volume elements (here we chose $n = 4$). For each volume element, we calculated the average stray field and bypass current, which were then used to determine the Hall voltage within that specific element (Fig. S4d and Fig. S4e for direct measurement, and Fig. S5d and Fig. S5e for inverse measurement). By summing up the contributions from all the volume elements, we obtained a more accurate estimation of the overall Hall voltage. This method, as the COMSOL picture illustrated in Fig. S6a and Fig. S6b for direct and inverse measurement, provides a significant improvement in

accuracy compared to a single-point estimation in COMSOL. Using this refined method, the estimated Hall voltage was found to be less than 3 nV under the applied current of 500 μA. Based on this value, the Hall effect-induced signal is calculated to be approximately 0.006 mΩ. In contrast, the experimentally measured signal is 0.22 mΩ, which is about two orders of magnitude larger than the calculated Hall effect contribution. This significant discrepancy strongly suggests that the Hall voltage induced by the combined effects of the stray field and bypass current has a negligible contribution to the measured signal in our experiments. Therefore, we conclude that the influence of the stray field can be effectively excluded from the observed signal.

Fig. S4

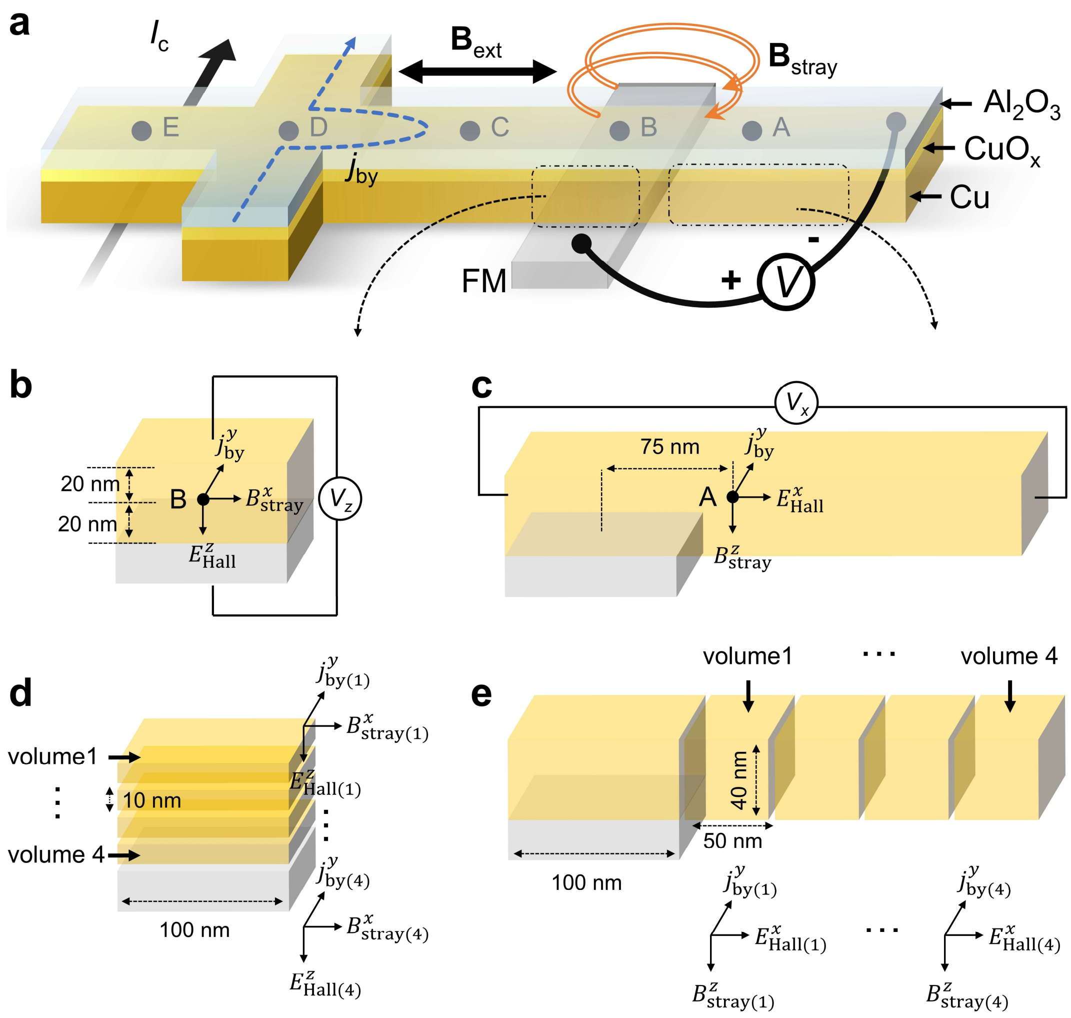

Fig. S4 | Hall voltage induced by stray field and bypass current in direct measurement. a, Schematic illustration of the stray field and bypass current in the direct measurement setup. The bypass current density (j_{by}) is represented by blue dashed curves, while the stray field ($\mathbf{B}_{stray}$) is shown in orange curves. Specific points along the centerline of the Cu_x nanowire, labeled as A, B, C, D, and E, are highlighted for further analysis. **b,** $\mathbf{B}_{stray}$ and j_{by} at the region near B point (above Cu/FM junction). A negative z-direction Hall electric field is induced, which can be detected by voltmeter. **c,** $\mathbf{B}_{stray}$ and j_{by} at the region near A point (on the right of Cu/FM junction). An x-direction Hall electric field is induced, which can be detected by voltmeter. **d, e,** The analysis method for COMSOL simulation. The bulk of Cu above FM (**d**) and that to the right of FM (**e**) is divided into 4 volume elements each. For each volume element, $\mathbf{B}_{stray}$ and j_{by} are simulated and averaged in each volume while the Hall voltage is calculated. This method provides a more accurate estimation by summing the contributions from all volume elements, compared to calculations based solely on a single point.

Fig. S5

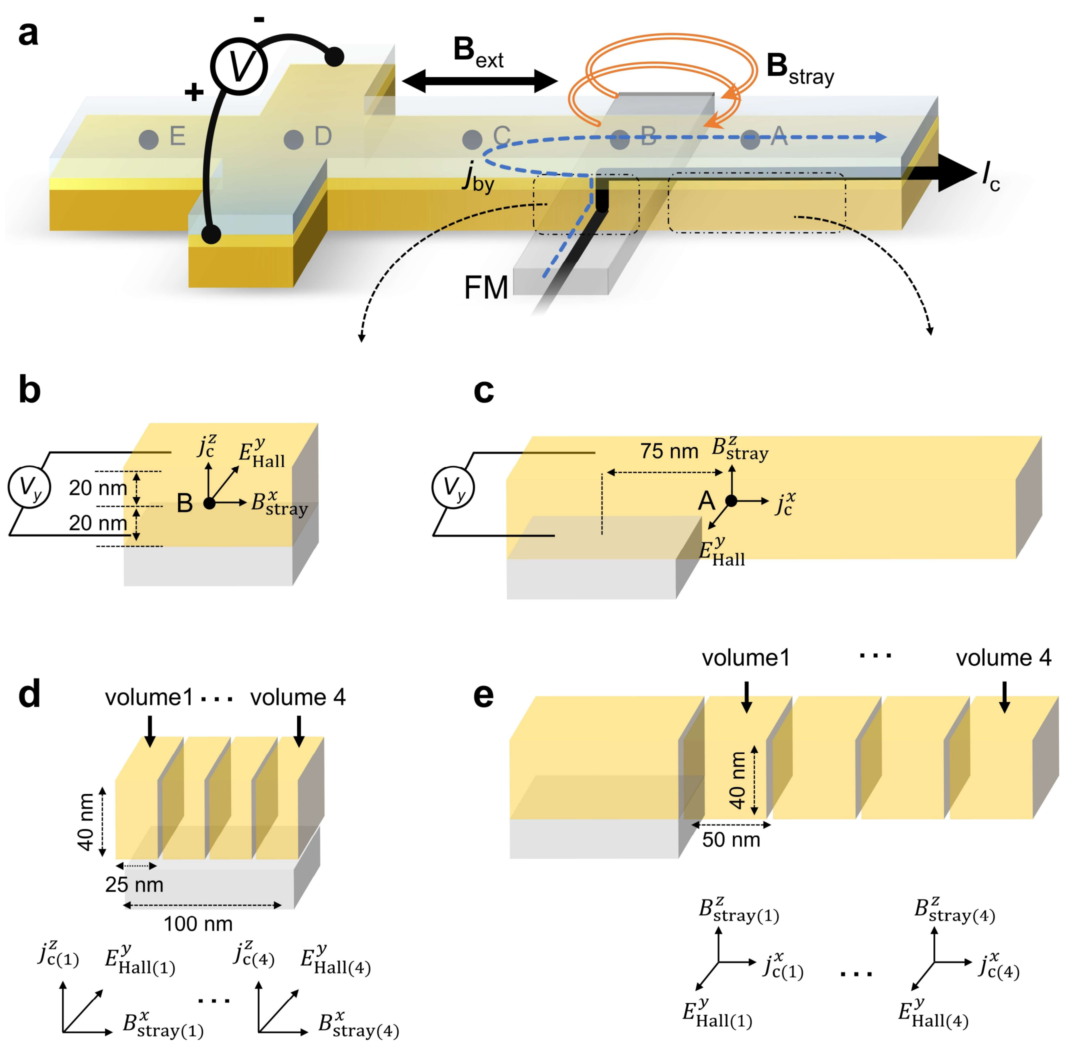

Fig. S5 | Hall voltage induced by stray field and bypass current in inverse measurement. a, Schematic illustration of the stray field and bypass current in the inverse measurement setup. The bypass current density (j_{by}) is represented by blue dashed curves, while the stray field ($\mathbf{B}_{stray}$) is shown in orange curves. Specific points along the centerline of the Cu_x nanowire, labeled as A, B, C, D, and E, are highlighted for further analysis. **b,** $\mathbf{B}_{stray}$ and j_{by} at the region near B point (above Cu/FM junction). A *y*-direction Hall electric field is induced, which can be detected by voltmeter. **c,** $\mathbf{B}_{stray}$ and j_{by} at the region near A point (on the right of Cu/FM junction). A *y*-direction Hall electric field is induced, which can be detected by voltmeter. **d, e,** The analysis method for COMSOL simulation. The bulk of Cu above FM (**d**) and that to the right of FM (**e**) is divided into 4 volume elements

each. For each volume element, $\mathbf{B}_{\mathrm{stray}}$ and j_{by} are simulated and averaged in each volume while the Hall voltage is calculated. This method provides a more accurate estimation by summing the contributions from all volume elements, compared to calculations based solely on a single point.

Fig. S6

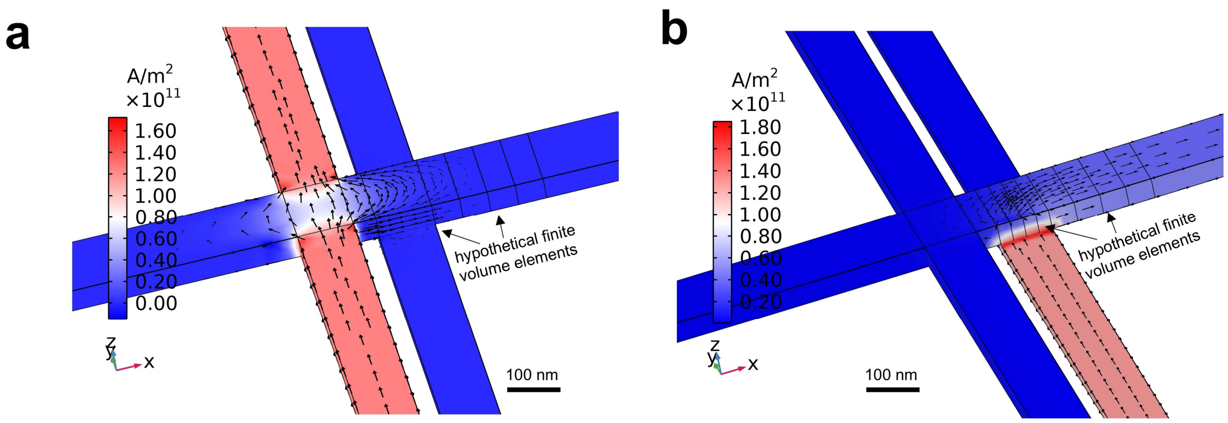

Fig. S6 | The illustration of COMSOL simulation on Hall voltage. a, b, COMSOL simulation of direct (**a**) and inverse (**b**) measurement setup. Hypothetical volume elements are depicted, while $\mathbf{B}_{\mathrm{stray}}$ is omitted for clarity. The color bars represent the current density. For each volume element, $\mathbf{B}_{\mathrm{stray}}$ and j_{by} are simulated and averaged, followed by the calculation of the Hall voltage within each volume element. The Hall voltages from all elements are then summed to determine the total Hall voltage, which contributes negligibly to the measured signal.

Section 4. Orbital accumulation measured in local transport structure

To explore solely the local distribution of orbital accumulation, we conducted the measurements exploiting a local transport structure[7] consisting of an $Al_2O_3/CuO_x/Cu$ nanowire (lays in the x-axis) on top of two separate FM nanowires (lays in the y-axis). The FM electrodes are patterned differently to have different switching fields. For the DOEE measurement (Fig. S7a), the nonequilibrium OAM are induced by charge current I_c via DOEE (charge-to-orbital conversion). In FMs, the orbital accumulation converts to spin accumulation (orbital-to-spin conversion) due to the spin-orbit coupling (SOC) of FMs and shifts the spin chemical potential in FMs. The difference of chemical potential in FM gives rise to the output voltage V. In contrast, in the inverse orbital Edelstein effect (IOEE) measurement (Fig. S7b), all processes are reversed. The nonequilibrium spin accumulation induced by charge current I_c convert to orbital accumulation due to SOC in FM (spin-to-charge conversion). The orbital accumulation converts to the charge current via IOEE (orbital-to-charge conversion), inducing a charge current and causing an output voltage of V. However, the spin current remains unaffected because of the lack of SOC in the $Al_2O_3/CuO_x/Cu$ nanowire. In both measurement configurations, the sweeping external magnetic field $\mathbf{B}_{ext}$ is applied along the easy axis of FMs (y-axis). The local direct ($R^{(0)}_{DOEE}$) and inverse ($R^{(0)}_{IOEE}$) orbital Edelstein resistance are defined as $R^{(0)} \equiv V/I_c$, where $R^{(0)}$ refers to both $R^{(0)}_{DOEE}$ and $R^{(0)}_{IOEE}$. Absolute value $|2\Delta R^{(0)}_{DOEE}|$ and $|2\Delta R^{(0)}_{IOEE}|$ refer to the overall change of $R^{(0)}_{DOEE}$ and $R^{(0)}_{IOEE}$. In local transport measurement, the orbital generator and detector are vertically separated in space, which allows the detection of pure vertical distribution of OAM.

Sample Fabrication

The samples for local transport measurement were fabricated on SiO_2/Si substrates through the electron beam lithography on polymethyl-methacrylate (PMMA) electron beam photoresist, develop, deposition, and lift-off processes. All devices share the same design with

specific variations explicitly noted. The 100 nm wide and 20 nm thick FM nanowire pairs, with a 50 nm gap and aligned along the same straight line, were deposited by electron beam deposition. The 200 nm wide and 40 nm thick Cu (t_{Cu}) nanowires were deposited by Joule heat evaporator (also t_{Cu} = 30, 50 nm were employed in Cu thickness dependence experiments, see Supplementary Section 8). Before the Cu deposition, an Ar-ion milling process was carefully conducted to the FM surface to obtain a clean Cu/FM interface. The samples were exposed to the atmosphere at room temperature for 10 minutes before Al_2O_3 deposition. The 15 nm thick Al_2O_3 capping layers to prevent Cu from further oxidation were only deposited on the Cu nanowires by electron beam deposition. As clearly shown in the following sections, the local transport structure exhibits a great feasibility for measuring orbital accumulation distribution, offering a method for probing other phenomena of orbital accumulation. Our local measurement experiments certify the presence of orbital accumulation and provide a measurement on vertical orbital distribution.

FM dependence

To seek the corroboration of orbital accumulation, we conducted FM dependence experiments by employing various FMs, such as $Co_{25}Fe_{75}$, $Co_{50}Fe_{50}$, and $Ni_{81}Fe_{19}$. Several samples with the same geometry are measured as shown in Fig. S7c ~ Fig. S7h, suggesting the following relationship: $|2\Delta R^{(0)}|(Co_{25}Fe_{75}) > |2\Delta R^{(0)}|(Co_{50}Fe_{50}) \gg |2\Delta R^{(0)}|(Ni_{81}Fe_{19})$. The variations in $R^{(0)}_{\mathrm{DOEE}}$ and $R^{(0)}_{\mathrm{IOEE}}$ are caused by the magnetization switching in the electrodes, as indicated by the single-headed arrow pairs in the figures. The FM dependence experiments consistently correlate with the nonlocal measurements. The results show a strong dependence on FMs, suggesting that the measured signals originate from orbital response instead of spin response.

Fig. S7

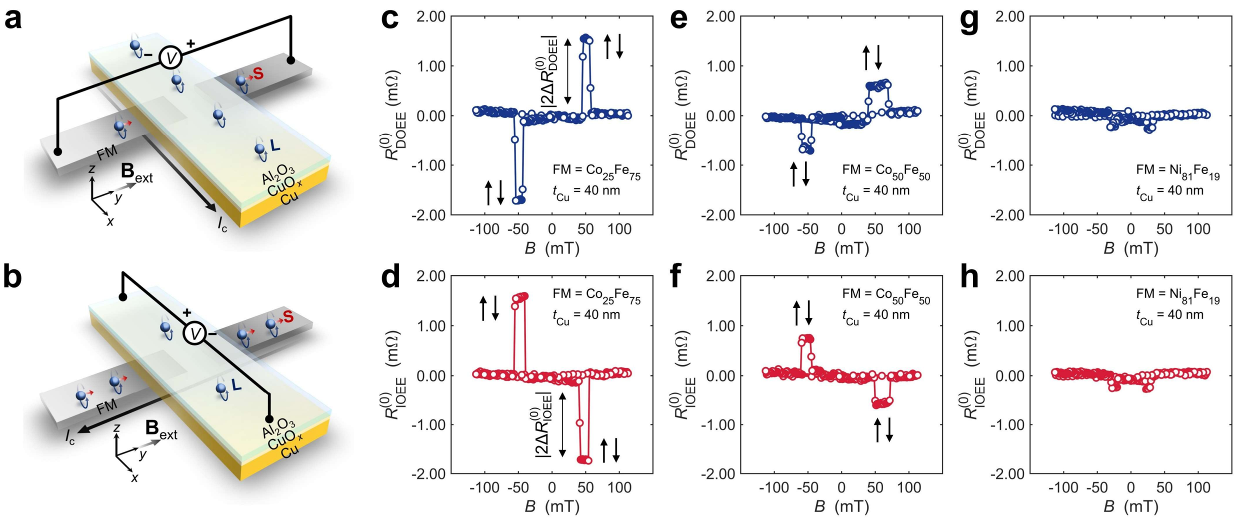

Fig. S7 | Schematic illustrations of local transport structures and verification of orbital accumulation through ferromagnetic materials dependence experiment. a, The local measurement configuration to observe DOEE (direct measurement). The nonequilibrium OAM (**L**) are generated at $Al_2O_3/CuO_x/Cu$ nanowire via DOEE. The orbital accumulation converts to spin accumulation (**S**) in FM and induced a local orbital response. **b,** The local measurement configuration to observe IOEE (inverse measurement). The spin accumulation (**S**) is induced by charge current, which further converts to orbital accumulation (**L**). The orbital accumulation then induces a charge current in $Al_2O_3/CuO_x/Cu$ nanowire via IOEE. **c, d,** The local measurements signals of DOEE (**c**) and IOEE (**d**), with the Cu thickness t_{Cu} = 40 nm and FM = $Co_{25}Fe_{75}$, showing that $|2\Delta R^{(0)}_{DOEE}| = |2\Delta R^{(0)}_{IOEE}|$ = 1.70 mΩ. The double-headed arrows represent the definition of $|2\Delta R^{(0)}_{DOEE}|$ and $|2\Delta R^{(0)}_{IOEE}|$. **e, f,** The local measurements result of DOEE (**e**) and IOEE (**f**), with the Cu thickness t_{Cu} = 40 nm and FM = $Co_{50}Fe_{50}$, showing that $|2\Delta R^{(0)}_{DOEE}| = |2\Delta R^{(0)}_{IOEE}|$ = 0.80 mΩ. **g, h,** The local measurements result of DOEE (**g**) and IOEE (**h**), with the Cu thickness t_{Cu} = 40 nm and FM = $Ni_{81}Fe_{19}$, whereas no OEE signals are shown. In **c** ~ **h**, the signals are globally offset to position their center at R = 0 Ω. The pairs of one-headed arrows in **c** ~ **h**

represent the magnetization configuration of FMs. The results show good agreement with Onsager's reciprocal relations.

Section 5. The role of spin current injected by electric current

Experimental Results

It is well established that charge-to-spin conversion is weak in systems composed of light elements due to the lack of strong spin-orbit coupling (SOC). Our device consists solely of Al_2O_3 and Cu (CuO_x) where the charge-to-spin conversion is expected to be negligible. We designed an additional experiment to provide clear validation. Here, we fabricated the new device by introducing an additional FM nanowire into the original device (see Fig. 1a in main text), as illustrated in Fig. S8a. This modification allows us to simultaneously measure the nonlocal OEE response and the nonlocal spin injection response within a single device.

The conventional nonlocal spin valve (NLSV) measurement configuration is shown in Fig. S8a. In this setup, an electric current I_c is applied through the lower FM nanowire, while the voltage signal V_{NLSV} is measured between the upper FM and the upper $Al_2O_3/CuO_x/Cu$ nanowire terminals. An external magnetic field is applied along the easy axis of FM. NLSV measurements were performed on two FM ($Ni_{81}Fe_{19}$ and $Co_{25}Fe_{75}$) at room temperature, with a separation distance of d_{NLSV} = ~400 nm (for both $Ni_{81}Fe_{19}$ and $Co_{25}Fe_{75}$ sample shown in Fig. S8) between the FMs and thickness of Cu is t_{Cu} = 40 nm. As shown in Fig. S8b and Fig. S8c, clear NLSV signals ($R_{NLSV} \equiv V_{NLSV}/I_c$) were observed in both $Ni_{81}Fe_{19}$ and $Co_{25}Fe_{75}$ devices, suggesting that spin current is injected into the $Al_2O_3/CuO_x/Cu$ nanowires.

The inverse orbital Edelstein effect measurement configuration is shown in Fig. S8d. In this setup, an electric current is applied through the lower FM nanowire, while the voltage signal V_{IOEE} is measured between the two ends of Cu_y terminals. The separation distance between Cu_y and lower FM nanowire is d = ~250 nm (for both $Ni_{81}Fe_{19}$ and $Co_{25}Fe_{75}$ sample shown in Fig. S8). However, for IOEE measurements, those two FMs exhibited starkly contrasting responses. In devices with FM = $Ni_{81}Fe_{19}$, no characteristic IOEE response was detected, while in $Co_{25}Fe_{75}$ devices, a clear IOEE signal was observed. This pronounced FM

dependence of the IOEE signal differs significantly from conventional spin responses, which are typically less sensitive to the choice of FM. Based on these results, we attribute the observed signal, as shown in the main text, predominantly to an orbital response, while the contribution of spin injection is minimal.

Sample Fabrication

Here we combine the nonlocal orbital transport device and nonlocal spin valve device in the signal sample (Fig. S8a and b). The samples were fabricated on SiO_2/Si substrates through the electron beam lithography on PMMA, develop, deposition, and lift-off processes. All devices share the same design with specific variations explicitly noted. The 100 nm wide and 20 nm thick FM (t_{FM}) nanowire pairs with a 400 nm separation distance (d_{NLSV} = ~400 nm for both $Ni_{81}Fe_{19}$ and $Co_{25}Fe_{75}$ sample, center-to-center) were deposited by electron beam deposition. The 40 nm thick Cu (t_{Cu}) nanowires were deposited by Joule heat evaporator. The Cu nanowires lying on the y-axis (Cu_y) are 100 nm wide, and the one lying on the x-axis (Cu_x) is 150 nm wide. The separation distance (center-to-center) between Cu_y and lower FM nanowire is d = 280 nm for $Ni_{81}Fe_{19}$ sample (Fig. S8a, d and e) and d = 300 nm for $Co_{25}Fe_{75}$ sample (Fig. S8f). Before the Cu deposition, an Ar-ion milling process was carefully conducted to the FM surface to obtain a clean Cu/FM interface. The samples were exposed to the atmosphere at room temperature for 10 minutes before Al_2O_3 deposition. The 15 nm thick Al_2O_3 capping layers to prevent Cu from further oxidation were only deposited on the Cu nanowires by electron beam deposition.

Fig. S8

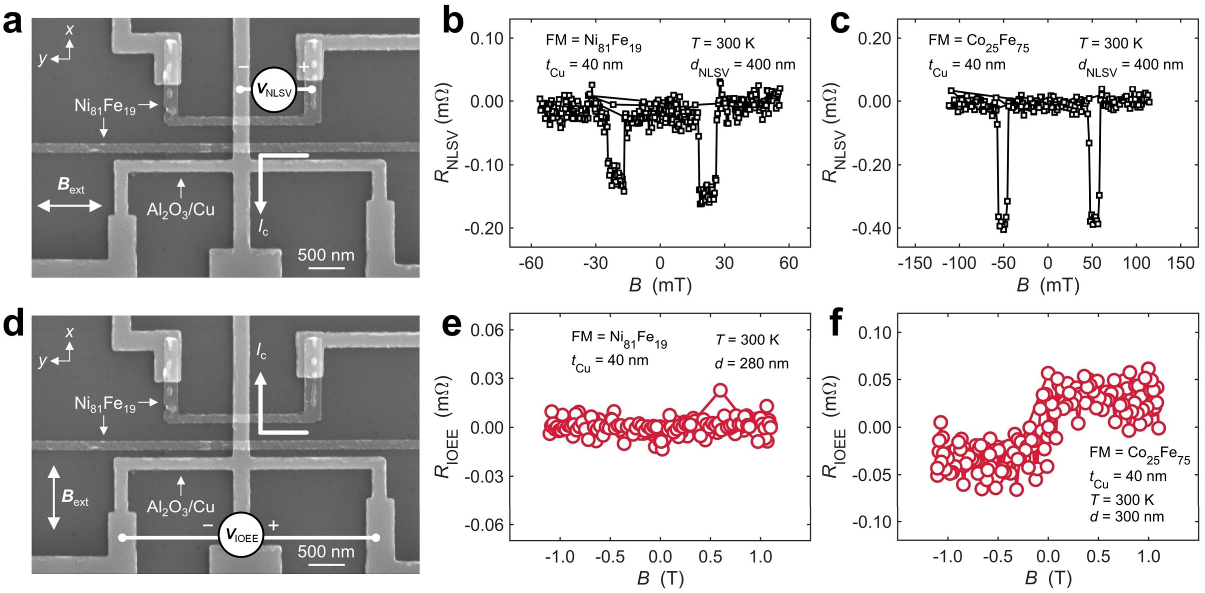

Fig. S8 | Nonlocal spin valve (NLSV) measurement and inverse OEE (IOEE) measurement at room temperature. a, The NLSV measurement configuration. The spin current, induced by charge current (I_c) in FM (lower one), is injected into the $Al_2O_3/CuO_x/Cu$ nanowire. The upper FM acts as a spin detector where an output voltage (V_{NLSV}) can be detected. **b,** The NLSV measurement results of device with $Ni_{81}Fe_{19}$ at room temperature. Here, R_{NLSV} is defined as $R_{NLSV} \equiv V_{NLSV}/I_c$. **c,** The NLSV measurement results of device with $Co_{25}Fe_{75}$ at room temperature. The NLSV devices in **b** and **c** share the same geometry. **d,** The nonlocal IOEE measurement configuration. The orbital accumulation is induced at the Cu/FM junction and injected into the $Al_2O_3/CuO_x/Cu$ nanowire. The lower $Al_2O_3/CuO_x/Cu$ horizontal nanowire acts as an orbital detector where an output voltage (V_{IOEE}) can be detected. **e,** The nonlocal IOEE measurement of device with $Ni_{81}Fe_{19}$ at room temperature (d = 280 nm). No characteristic signal is observed. **f,** The nonlocal IOEE measurement of device with $Co_{25}Fe_{75}$ at room temperature (d = 300 nm). A typical nonlocal IOEE signal is observed.

Section 6. The nonlocal orbital distribution model which considers bypass current

The COMSOL simulation of bypass current density

We first performed the COMSOL simulation to study the flow of charge current density, as shown in Fig. S9a. The y-component of bypass current density j_{by}^{y} is evaluated (averaged value) over the cross section of Cu$_x$ nanowire above the center of FM nanowires at various distance. j_{by}^{y} as a function of d is summarized in Fig. S9b. The selection of d was based on practical devices. Assuming the j_{by}^{y} decays exponentially as $j_{\mathrm{by}}^{y} \propto \exp\left(-\frac{d}{\lambda_{\mathrm{by}}}\right)$, the fitting yields a decay length of bypass current $\lambda_{\mathrm{by}} \cong 47$ nm (Fig. S2c). However, fitting the results of experimental nonlocal transport measurement to $\Delta R_{\mathrm{NL}} \propto \exp\left(-\frac{d}{\lambda_{\mathrm{NL}}}\right)$ gives rise to a lateral decay length $\lambda_{\mathrm{NL}} \cong 110$ nm, as shown in the Fig. S9c and Fig. S9d. The stimulated results of bypass current show a decay two times faster than observed signals and suggest that the bypass current is not the main origin of the signals. Therefore, we believe the signals are the combination of bypass effect of charge current and the lateral distribution of OAM.

The analytical model of nonlocal distribution of orbital accumulation considering bypass current

We hereby analyze λ_{o} by considering the combined contribution of the bypass effect of charge current and the nonlocal distribution of orbital accumulation. We assume the y-component charge current density is uniform in the Cu cross region, which can be expressed as:

$$j_{\mathrm{c}}^{y}(0) = \frac{I_{\mathrm{c}}\xi_{\mathrm{by}}}{W_y t_{\mathrm{Cu}}}. \qquad (\mathrm{S6}-1)$$

where I_{c} is the applied current, ξ_{by} is a shutting constant, W_y is the width of Cu$_y$ nanowire, t_{Cu} is the thickness of the Cu. To provide a simple picture of the charge bypass and orbital decay joint effect, we consider an effective one-dimensional model, where the y-component of bypass

current density j_{by}^{y} is solely a function of the distance x from the center of Cu_y nanowire (as shown in the Fig. S3a). Its magnitude can be written as[4,5]:

$$j_{\mathrm{by}}^{y}(x) = j_{\mathrm{c}}^{y}(0)\exp\left(-\frac{\pi x}{W_x}\right), \qquad (\mathrm{S6}-2)$$

where W_x is the width of the Cu_x nanowire. ξ_{by} can be self-consistently determined as follows:

$$2t_{\mathrm{Cu}}\int_0^{+\infty} j_{\mathrm{by}}^{y}(x)dx = 2t_{\mathrm{Cu}}\int_0^{+\infty}\frac{I_{\mathrm{c}}\xi_{\mathrm{by}}}{W_y t_{\mathrm{Cu}}}\exp\left(-\frac{\pi x}{W_x}\right)dx = I_{\mathrm{c}},$$

$$\xi_{\mathrm{by}} = \left(\frac{\pi W_y}{2W_x}\right). \qquad (\mathrm{S6}-3)$$

Here, the influence of FM (because the resistance of FM nanowire is much more significant than that of Cu nanowire) and the difference in width between Cu_y and Cu_x are ignored. Factor 2 is included since the bypass effect has two sides. The generated orbital accumulation a_{o}^{x} can be induced by j_{by}^{y} at x as following (y-polarized OAM cancel out each other, as shown in the Fig. S3b):

$$a_{\mathrm{o}}^{\mathrm{g}}(x) = q_{ICO}t_{\mathrm{CuO}}j_{\mathrm{by}}^{y}(x) \qquad (\mathrm{S6}-4)$$

where t_{CuO} is the interfacial oxidation thickness, and q_{ICO} is the interfacial charge-to-orbital conversion efficiency similar to the spin Rashba counterpart in topological insulator[8–10]. Subsequently, $a_{\mathrm{o}}^{\mathrm{g}}(x)$ undergoes a decay from x to d (from its generation location to the center of FM). Thus, the contribution from $a_{\mathrm{o}}^{\mathrm{g}}(x)$ to the orbital accumulation at d can be expressed as following if we assume it decays exponentially (as shown in the Fig. S3c):

$$\Delta\alpha_{\mathrm{o}}^{d}(d,x) = a_{\mathrm{o}}^{\mathrm{g}}(x)\exp\left(-\frac{d-x}{\lambda_{\mathrm{o}}}\right) \qquad (\mathrm{S6}-5)$$

where λ_{o} is the lateral decay length of orbital accumulation. The overall orbital accumulation α_{o}^{x} at d is an integration of $\Delta\alpha_{\mathrm{o}}^{d}(d,x)$ over the area from the center of Cu cross to the center of FM given by:

$$
\begin{aligned}
\alpha_{\mathrm{o}}^{x}(d) &= \int_{0}^{d}\int_{-W_x/2}^{+W_x/2} \Delta\alpha_{\mathrm{o}}^{d}(d,x)\,dx\,dy \\
&= \int_{0}^{d}\int_{-W_x/2}^{+W_x/2} q_{ICO}\,t_{\mathrm{CuO}}\frac{I_{\mathrm{c}}}{t_{\mathrm{Cu}}}\frac{\pi}{2W_x}\exp\left(-\frac{\pi x}{W_x}\right)\exp\left(-\frac{d-x}{\lambda_{\mathrm{o}}}\right)dx\,dy \\
&= \frac{\pi I_{\mathrm{c}} q_{ICO} t_{\mathrm{CuO}}}{2t_{\mathrm{Cu}}\left(\frac{1}{\lambda_{\mathrm{o}}}-\frac{\pi}{W_x}\right)}\left[\exp\left(-\frac{\pi d}{W_x}\right)-\exp\left(-\frac{d}{\lambda_{\mathrm{o}}}\right)\right]. \qquad (\mathrm{S6}-6)
\end{aligned}
$$

The nonlocal voltage generated from the chemical potential is proportional to $\alpha_{\mathrm{o}}^{x}(d)$. Suppose $V = A_{\mathrm{o}}\alpha_{\mathrm{o}}^{x}(d)$, where the coefficient A_{o} depends on the thickness of the Cu layer, the orbital polarization of the FM, the interfacial orbital transmission efficiency and the area of the FM/Cu junction, the nonlocal direct orbital Edelstein resistance can then be described as:

$$
\begin{aligned}
2\Delta R_{\mathrm{DOEE}} = \frac{V}{I_{\mathrm{c}}} &= \frac{\pi q_{ICO} t_{\mathrm{CuO}}}{2t_{\mathrm{Cu}}\left(\frac{1}{\lambda_{\mathrm{o}}}-\frac{\pi}{W_x}\right)}\left[\exp\left(-\frac{\pi d}{W_x}\right)-\exp\left(-\frac{d}{\lambda_{\mathrm{o}}}\right)\right] \\
&= \frac{A}{\left(\frac{1}{\lambda_{\mathrm{o}}}-\frac{\pi}{W_x}\right)}\left[\exp\left(-\frac{\pi d}{W_x}\right)-\exp\left(-\frac{d}{\lambda_{\mathrm{o}}}\right)\right], \qquad (\mathrm{S6}-7)
\end{aligned}
$$

where $2\Delta R_{\mathrm{DOEE}}$ represents the overall change of nonlocal direct orbital Edelstein effect, and A is the fitting parameter containing the orbital Edelstein length q_{ICO} and the orbital absorption efficiency A_{o}.

Fig. S9

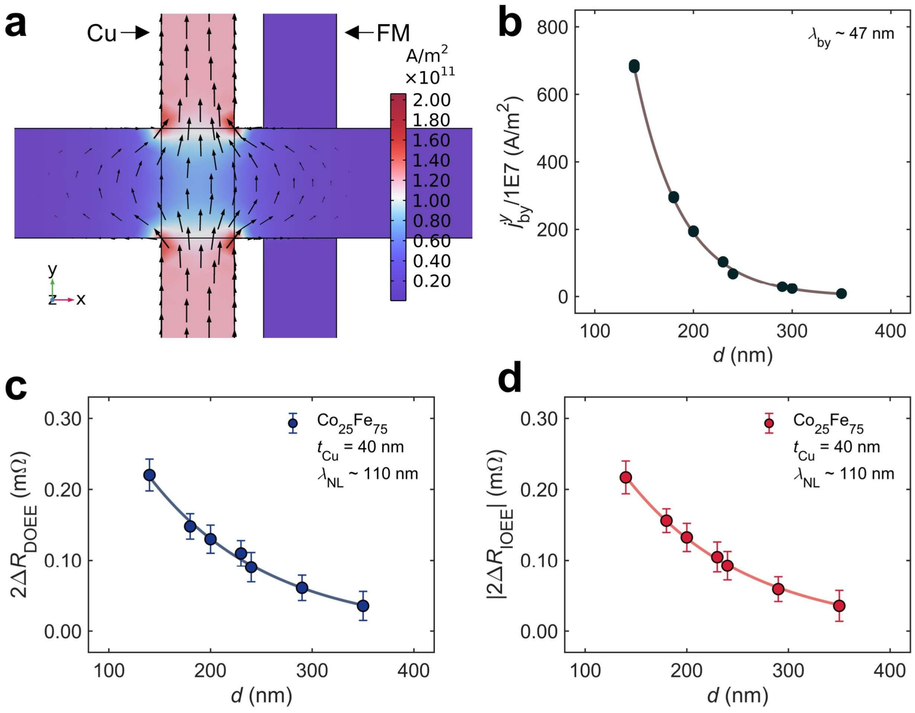

Fig. S9 | The analysis of bypass current. a, The COMSOL simulation for j^y_{by} in our nonlocal transport structure, where FM nanowire (right) is under Cu nanowire (left). The color bar denotes the magnitude of the y-component current density (j^y_{by}). The black arrows denote the flow of the charge current density (j_{c}) while their sizes indicate the relative magnitude. The color bar represents the current density. **b,** The exponential fitting ($j^y_{\mathrm{by}} = A\exp(-d/\lambda_{\mathrm{by}})$) results of j^y_{by} (the average over the cross-sectional area) to d, suggesting a decay length of bypass current of about 47 nm. **c, d,** The exponential fitting ($\Delta R_{\mathrm{NL}} \propto \exp(-d/\lambda_{\mathrm{NL}})$) result of $2\Delta R_{\mathrm{DOEE}}$ (**c**) and $|2\Delta R_{\mathrm{IOEE}}|$ (**d**) measured by nonlocal transport structure, indicating a characteristic length of nonlocal resistance about 110 nm. The distinction implies that bypass current is not the main origin of our nonlocal signal. The error bars indicate the standard derivation of raw data (R_{DOEE} and R_{IOEE}) after the magnetization is saturated.

Section 7. The analysis of the angle dependence

Here, we discuss the deviation between experimental data and the cosine curves shown in Fig. 2c and 2d in the main text. The deviation can be explained if we make the following two assumptions:

1. Angular dependence is governed by the direction of magnetization **m** rather than the external magnetic field $\mathbf{B}_{\text{ext}}$. Thus, $2\Delta R_{\text{DOEE}}$ and $2\Delta R_{\text{IOEE}}$can be written as:

$$2\Delta R_{\text{DOEE}} = |2\Delta R_{\text{IOEE}}| = f_1 \cos(\phi_m) \qquad (\text{S}7-1)$$

   where the ϕ_m denotes the in-plane angle of **m** to the *x*-axis.

2. Ferromagnet (FM) has an easy axis along the *y*-axis. Therefore, we have:

$$F = -\mathbf{B}_{\text{ext}} \cdot \mathbf{m} - K(\mathbf{m} \cdot \hat{y})^2, \qquad (\text{S}7-2)$$

   where *F* denotes the free energy density of FM and *K* denotes the in-plane easy-axis anisotropy along the *y*-axis.

Thus, Assumption 1 implies that $2\Delta R_{\text{OEE}}$ depends on ϕ_m rather than Φ (the angle of $\mathbf{B}_{\text{ext}}$ from the *y*-axis). When $\mathbf{B}_{\text{ext}}$ is sufficiently strong, ϕ_m will be close to Φ, but there will be a slight deviation between ϕ_m and Φ, which decays with increasing $\mathbf{B}_{\text{ext}}$. The in-plane magnetic anisotropy will determine the amount of deviation. In Assumption 2, the difference between ϕ_m and Φ can be determined by minimizing *F* with respect to ϕ_m. For minimization, we rewrite Eq. S7 − 2 as follows:

$$F = -\mathbf{B}_{\text{ext}} \cdot \mathbf{m} - K(\mathbf{m} \cdot \hat{\mathbf{y}})^2 = -B_{\text{ext}} \cos(\phi_m - \Phi) - K \sin^2 \phi_m \,. \qquad (\text{S}7-3)$$

From $\partial F/\partial \phi_m = 0$, we obtain:

$$B_{\text{ext}} \sin(\phi_m - \Phi) = K \sin 2\,\phi_m. \qquad (\text{S}7-4)$$

When B_{ext} is much larger than *K*, we may take:

$$\phi_m = \Phi + \delta\phi_m. \qquad (\text{S}7-5)$$

By inserting Eq. S7 − 5 into Eq. S7 − 3, one obtains:

$$\delta\phi_m = \frac{K}{B_{\text{ext}}}\sin 2\Phi + O\left(\frac{K}{B_{\text{ext}}}\right)^2. \qquad (\text{S7}-6)$$

From Eq. S7-1, S7-5 and S7-6, one obtains:

$$2\Delta R_{\text{DOEE}} = |2\Delta R_{\text{IOEE}}| = f_1 \cos\left(\Phi + \frac{K}{B_{\text{ext}}}\sin 2\Phi\right) \qquad (\text{S7}-7)$$

which can be modified as

$$\begin{aligned} 2\Delta R_{\text{DOEE}} = |2\Delta R_{\text{IOEE}}| &= f_1 \cos\left(\Phi + \frac{|f_2|}{2f_1}\sin 2\Phi\right) \\ &= f_1 \cos\left(\Phi - \frac{f_2}{f_1}\sin\Phi\cos\Phi\right) \\ &\approx f_1\left(\cos\Phi + \frac{f_2}{f_1}\sin^2\Phi\cos\Phi\right) \\ &= f_1\cos\Phi + f_2\sin^2\Phi\cos\Phi \qquad (\text{S7}-8) \end{aligned}$$

if the ratio $|f_2|/f_1$ satisfies the following relation,

$$\frac{|f_2|}{2f_1} = \frac{K}{B_{\text{ext}}}. \qquad (\text{S7}-9)$$

Fitting the deviation value $2\Delta R_{\text{OEE}} - 2\Delta R_{\text{fit}}$ to Eq. S7 − 8 (i.e., $2\Delta R_{\text{OEE}} - f_1\cos\Phi = f_2\sin^2\Phi\cos\Phi$) shows a high consistency, as shown in Fig. S10a and Fig. S10b, where K is estimated as 0.20 ~ 0.26 T with Eq. S7 − 9. The value is reasonable for 100 nm wide and 20 nm thick $Co_{25}Fe_{75}$ nanowires, implying that the deviation may originate from the magnetization anisotropy of FM.

In addition to magnetization anisotropy, orbital anisotropy may also contribute to the deviation via the orbital-spin conversion process. Eq. S7 − 8 can be rewritten as:

$$2\Delta R_{\text{DOEE}} = |2\Delta R_{\text{IOEE}}| = (f_1 + f_2)\mathbf{m}\cdot\mathbf{B} - f_2(\mathbf{m}\cdot\mathbf{B})^2\mathbf{m}\cdot\mathbf{B}, \qquad (\text{S7}-10)$$

where the f_2 term is a high-order contribution in the orbital-spin conversion that comes from the orbital anisotropy. Similar orbital anisotropy was reported in an orbital torque study, where the torque efficiency shows a sizable high-order term in the angular dependence of the orbital torque generated by OEE[11]. As both mechanisms may contribute to the f_2 term, the observed slight deviation in the angular dependence is reasonable.

Fig. S10

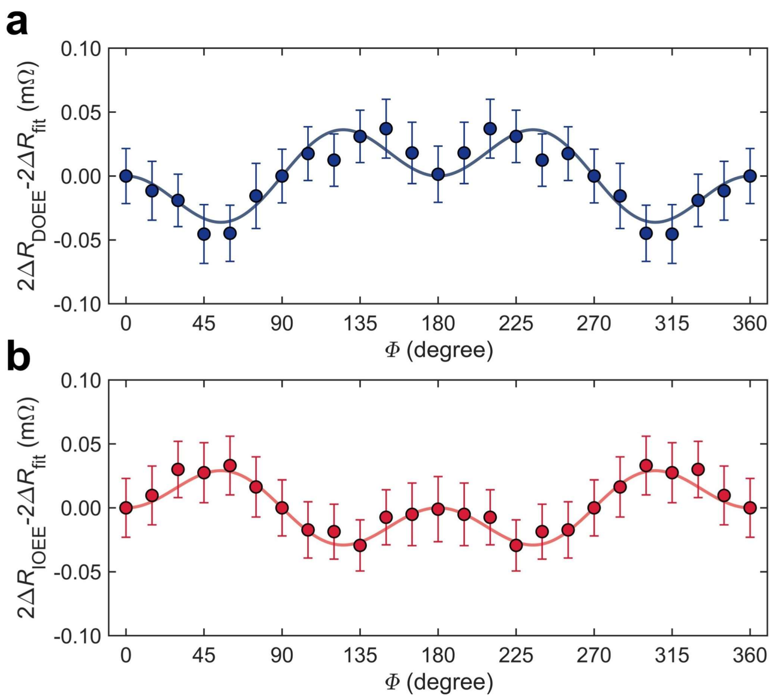

Fig. S10 | The deviation of angle dependence data. a, b, The fitting of the direct measurement (**a**) and inverse measurement (**b**) data to Eq. $S7-8$, suggesting the magnetization anisotropy may lead to the deviation in Fig. 2c and 2d (in main text). The solid curves represent the fitting. The error bars indicate the standard derivation of raw data (R_{DOEE} and ΔR_{IOEE}) after the magnetization is saturated.

Section 8. Cu thickness dependence measured in local transport structure

We conducted experiments to examine the dependency of Cu thickness by using local transport measurement. The samples measured share the same geometry except the Cu thickness. $R^{(0)}_{\mathrm{DOEE}}$ and $R^{(0)}_{\mathrm{IOEE}}$ for Cu thicknesses of 30 nm, 40 nm, and 50 nm are shown in Fig. S11a ~ Fig. S11f, while $2\Delta R^{(0)}_{\mathrm{DOEE}}$ and $2\Delta R^{(0)}_{\mathrm{IOEE}}$ are summarized in Fig. S11g and Fig. S11h. The signals $2\Delta R^{(0)}_{\mathrm{DOEE}}$ and $2\Delta R^{(0)}_{\mathrm{IOEE}}$ decrease as Cu thickness increases because of the longer transport distance, which is consistent with the results obtained in nonlocal measurements. By assuming the orbital accumulation distribution decays exponentially in local transport, the signal can be expressed as $|2\Delta R^{(0)}_{\mathrm{DOEE}}| = |2\Delta R^{(0)}_{\mathrm{IOEE}}| = A^{(0)}_{\mathrm{Cu}} \exp(-t_{\mathrm{cu}}/\lambda^{z}_{\mathrm{o}})$. Fitting the data to the equation yields that $\lambda^{z}_{\mathrm{o}} \sim 36$ nm, which is consistent with the value (~ 25 nm) obtained from the nonlocal Cu thickness dependence experiment in main text.

Fig. S11 Local measurement of Cu thickness dependence

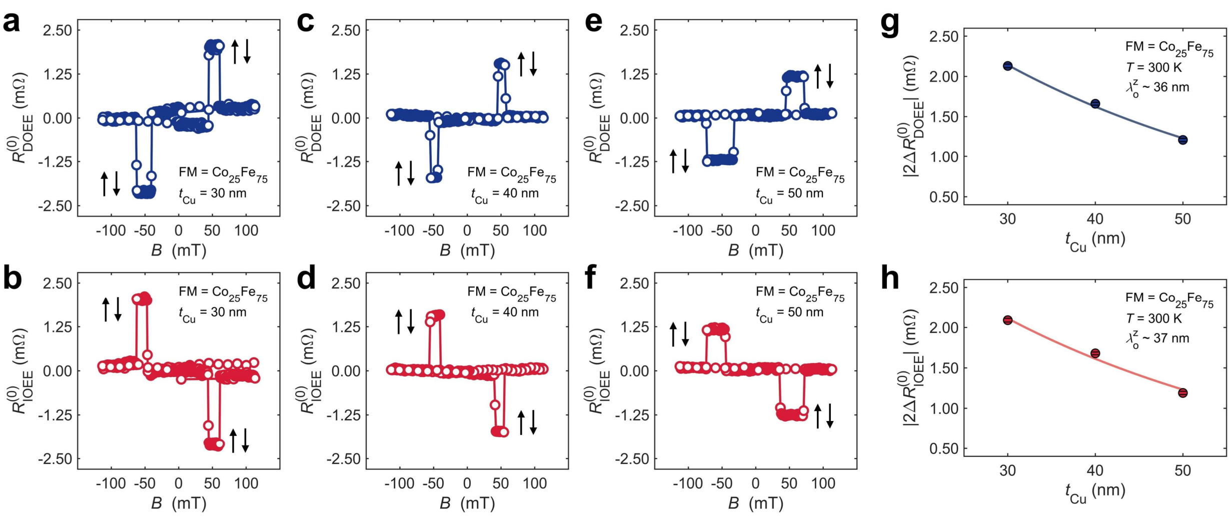

Fig. S11 | Cu thickness dependence experiment exploiting local transport structures. **a, b** The local transport measurement results of $R^{(0)}_{\mathrm{DOEE}}$ and $R^{(0)}_{\mathrm{IOEE}}$ with Cu thickness of 30 nm, showing that$|2\Delta R^{(0)}_{\mathrm{DOEE}}| = |2\Delta R^{(0)}_{\mathrm{IOEE}}| = 2.20$ mΩ. **c, d** The results of $R^{(0)}_{\mathrm{DOEE}}$ and $R^{(0)}_{\mathrm{IOEE}}$ with Cu thickness of 40 nm, showing that$|2\Delta R^{(0)}_{\mathrm{DOEE}}| = |2\Delta R^{(0)}_{\mathrm{IOEE}}| = 1.70$ mΩ. **e, f** The results of $R^{(0)}_{\mathrm{DOEE}}$

and $R^{(0)}_{\mathrm{IOEE}}$ with Cu thickness of 50 nm, showing that$|2\Delta R^{(0)}_{\mathrm{DOEE}}| = |2\Delta R^{(0)}_{\mathrm{IOEE}}| = 1.20$ mΩ. The results are consistent with nonlocal measurement results and show good agreement with Onsager's reciprocal relations. In **a** ~ **f**, the signals are globally offset to position their center at $R = 0$ Ω. The pairs of one-headed arrows in **a** ~ **f** represent the magnetization configuration of FMs. **g, h,** $|2\Delta R^{(0)}_{\mathrm{DOEE}}|$ and $|2\Delta R^{(0)}_{\mathrm{IOEE}}|$ with various Cu thickness. The error bars indicating the standard derivation of R after the magnetization is saturated are smaller than the range of markers. The solid curves demonstrate the fitting of data to the exponential decay equation, showing a local decay length of orbital accumulation ~ 36 nm. The measurements are performed under room temperature. The results show good agreement with Onsager's reciprocal relations.

Section 9. Temperature dependence measured in local transport structure

Experimental results for nonlocal DOEE and IOEE measurements on sample A (*d* = 140 nm, t_{Cu} = 40 nm, FM = $Co_{25}Fe_{75}$) as a function of temperature are presented in Fig. S12a and Fig. S12b, respectively.

Fig. S12

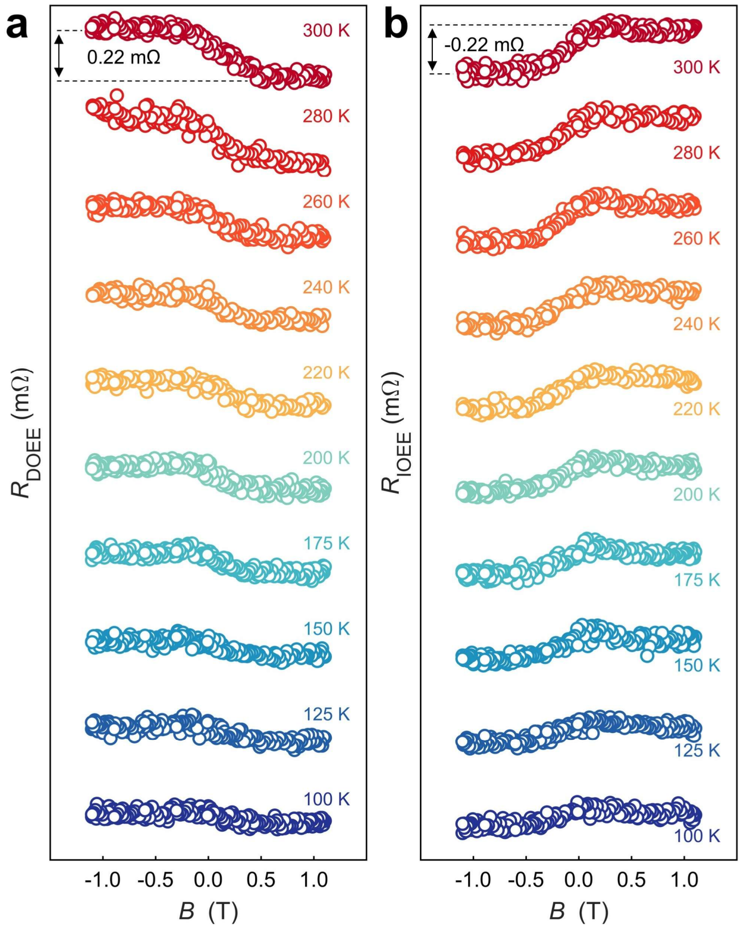

Fig. S12 | Temperature dependence measurement exploiting nonlocal transport structures (FM = $Co_{25}Fe_{75}$). a, b The results of R_{DOEE} (**a**) and R_{IOEE} (**b**) at various temperatures measured in sample A, respectively. As temperature decreases, the orbital response diminishes. The temperature dependence of nonlocal orbital transport is consistent with the local one (Fig. S13) and shows good agreement with Onsager's reciprocal relations.

We explored the temperature dependence of samples with 40 nm thick Cu employing $Co_{25}Fe_{75}$ through local OEE measurement. The results at different temperatures are shown in Fig. S13a and b. A consistent reduction in $R^{(0)}_{\mathrm{DOEE}}$ and $R^{(0)}_{\mathrm{IOEE}}$ with decreasing temperature was observed, aligning with results from nonlocal measurement experiments, showing a special temperature dependence of orbital accumulation distribution.

Fig. S13

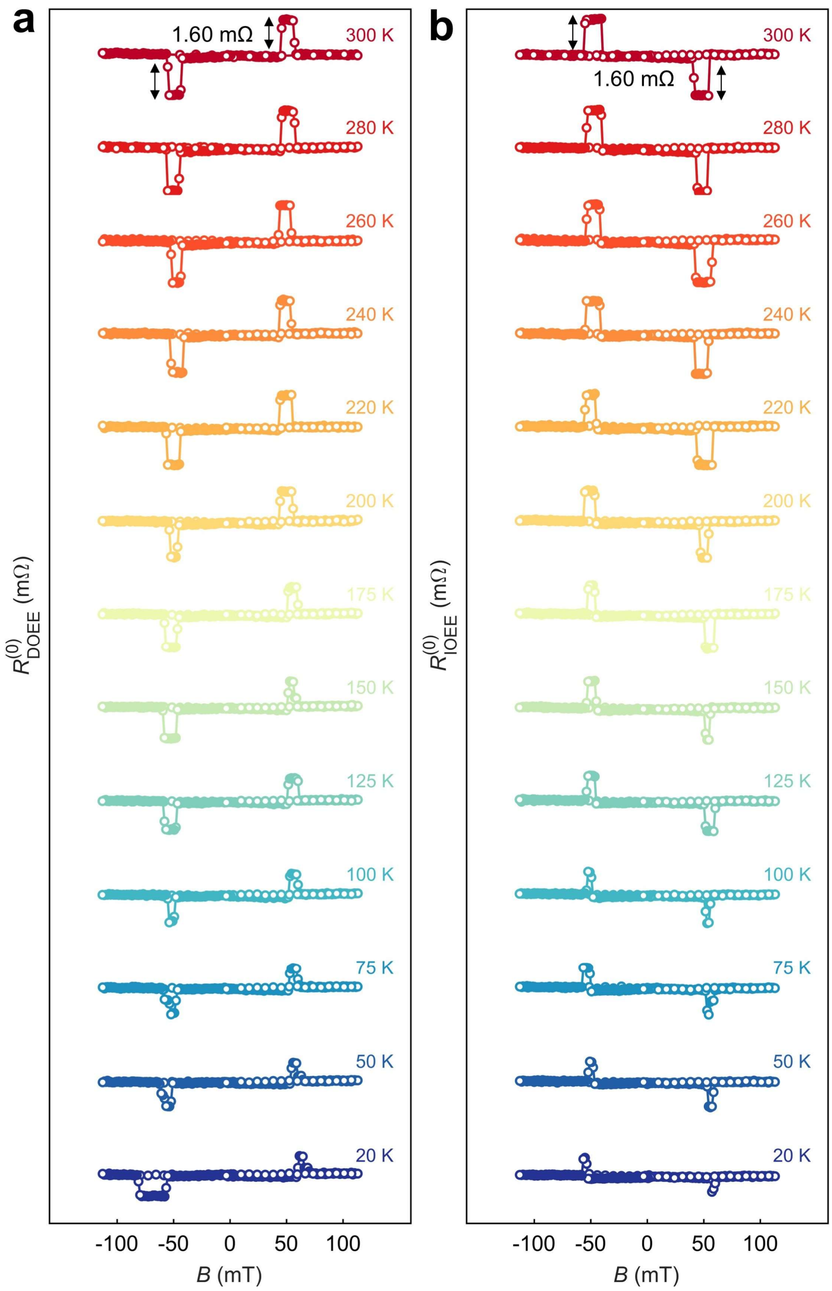

Fig. S13 | Temperature dependence experiment exploiting local transport structures (FM = $Co_{25}Fe_{75}$). a, b The results of R_{DOEE} (**a**) and R_{IOEE} (**b**) at various temperatures for Cu thickness of 40 nm, respectively. The temperature dependence of local orbital transport is consistent with nonlocal measurement results and shows good agreement with Onsager's reciprocal relations.

Here, the nonlocal and local IOEE temperature dependence measurement results ($2\Delta R_{\mathrm{IOEE}}$ and $\left|2\Delta R_{\mathrm{IOEE}}^{(0)}\right|$ in Fig. S12b and Fig. S13b, respectively) are further summarized in the Fig. S14.

Fig. S14

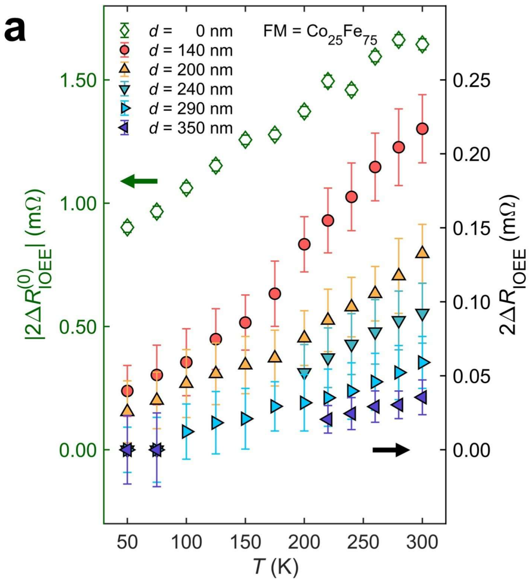

Fig. S14 | Temperature dependence of IOEE. a, The results of $|2\Delta R_{\mathrm{IOEE}}^{(0)}|$ and $2\Delta R_{\mathrm{IOEE}}$ as a function of temperature. The green open diamonds indicate the $|2\Delta R_{\mathrm{IOEE}}^{(0)}|$. The circles and triangles markers filled with other color indicate the $|2\Delta R_{\mathrm{IOEE}}|$. The results are measured in samples with t_{Cu} = 40 nm and FM = $Co_{25}Fe_{75}$. The error bars indicate the standard derivation of R in the plateau.

Furthermore, we studied the temperature dependence of DOEE (Fig. S15a) and IOEE (Fig. S15b) in the $Ni_{81}Fe_{19}$ devices. In all temperature points, no characteristic signal is shown.

Fig. S15

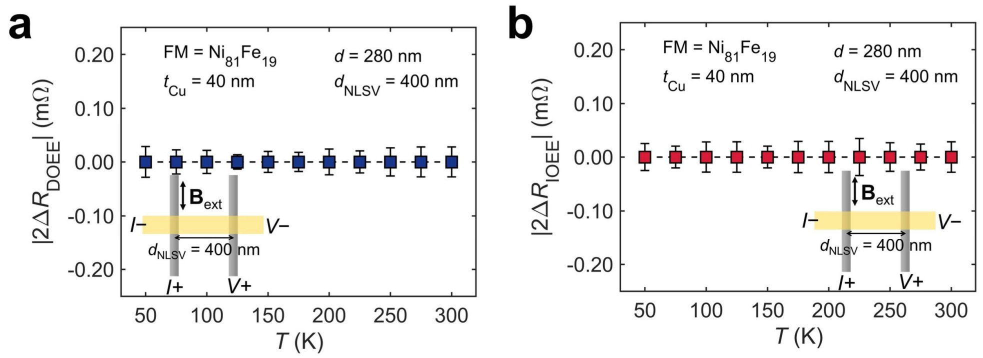

Fig. S15 | Temperature dependence of DOEE and IOEE measured in FM = $Ni_{81}Fe_{19}$ device. a, $|2\Delta R_{DOEE}|$ as a function of temperature. **b,** $|2\Delta R_{IOEE}|$ as a function of temperature. The results are measured in sample with t_{Cu} = 40 nm and FM = $Ni_{81}Fe_{19}$. The dashed line represents the position $2\Delta R = 0$. The error bars indicate the standard derivation of R in the plateau.

The IOEE data obtained from different devices at the same temperature were fitted using Eq. 1 to obtain the temperature-dependent λ_o (λ_o is the only fitting parameter), with the fitting process shown in Fig. S15.

Fig. S16

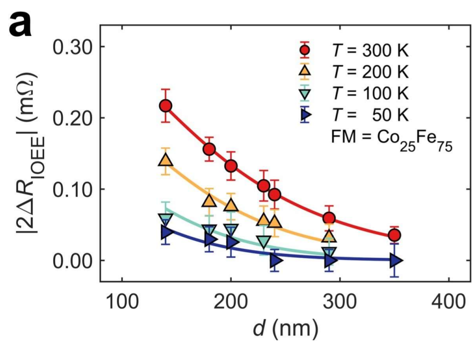

Fig. S16 | The fitting of nonlocal IOEE data. a, The fitting of the temperature-dependent $2\Delta R_{IOEE}$ data with Eq. (1). Here, t_{Cu} = 40 nm and FM = $Co_{25}Fe_{75}$. The solid curves represent the fitting. The error bars indicate the standard derivation of ΔR_{IOEE} after the magnetization is saturated.

Section 10. Control experiment with Au/Cu nanowire

Experimental Results

As a control experiment, the samples composed of Au, Cu and $Co_{25}Fe_{75}$ were measured. The control samples share the identical design as the experimental samples (Fig. 1a and 1b in main text). However, a 5 nm thick Au capping layer was in-situ deposited by Joule heat deposition immediately after Cu deposition. Au is highly stable at room temperature, effectively preventing Cu oxidation. With a full *d*-shell, Au does not provide necessary orbitals for orbital hybridization, hence unlikely to support a strong orbital Rashba effect. Although a spin Rashba effect (SRE) has been reported at the Au/Cu interface, this effect is negligible at room temperature[12].

The control experiment results are presented in Fig. S17. The samples share the same design as experiment samples shown in the main text. In the Au/Cu samples, no measurable nonlocal signal was observed at room temperature (Fig. S17a and Fig. S17b, black squares; Fig. S17c, open triangles), in contrast to the clear orbital response in $Al_2O_3/CuO_x/Cu$ samples (Fig. 1c and 1d in main text). At 50 K, a small signal (~ 0.02 mΩ) appeared in the Au/Cu samples (Fig. S17a and Fig. S17b, green circles; Fig. S17c, close triangles with d_{SRE} = 300 nm), whereas the signal was negligible in the Cu oxidized samples (Fig. 4a in main text). Moreover, the sign of the low-temperature signal in Au/Cu samples (green circles in Fig. S17a and Fig. S17b) is opposite to that in the Cu oxidized samples (black squares in Fig. 1c and 1d).

Taken together, these three observations—the absence of signal at room temperature, the distinct temperature dependence, and the opposite sign—demonstrate that the OEE is suppressed in the Au/Cu control devices. Instead, the signals appeared at 50 K are likely due to direct and inverse spin Rashba effect (DSRE and ISRE) at the Au/Cu interface, consistent with previous reports[12]. Those findings demonstrated that in a heterostructure without CuO_x

and other sources with p, d orbitals for hybridization, the sizable signal coming from orbital transport diminishes.

Sample Fabrication

The samples were fabricated on SiO_2/Si substrates through the electron beam lithography on PMMA, develop, deposition, and lift-off processes. All devices share the same design with specific variations explicitly noted. The 100 nm wide and 20 nm thick FM (t_{FM}) nanowires were deposited by electron beam deposition. The 40 nm thick Cu (t_{Cu}) nanowires were deposited by Joule heat evaporator. Before the Cu deposition, an Ar-ion milling process was carefully conducted to the FM surface to obtain a clean Cu/FM interface. The Cu nanowires lying on the y-axis (Cu_y) are 100 nm wide, and the one lying on the x-axis (Cu_x) is 150 nm wide. After the Cu deposition, a 5 nm thick Au capping layer was immediately in-situ deposited on Cu to prevent Cu from oxidation. The separation distance (center-to-center) between Au/Cu_y and FM nanowire is d_{SRE} = 300 nm.

Fig. S17

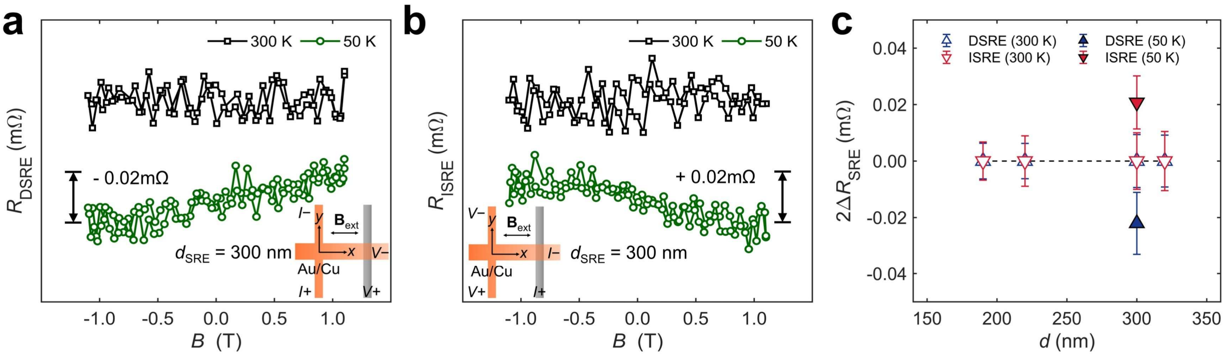

Fig. S17 | The measurement results in Au/Cu devices. a, The results measured with direct measurement configuration. At room temperature, no signal was detected (black squares). At 50 K, a typical signal was observed which is attributed to the direct spin Rashba effect (DSRE). **b,** The results measured with inverse measurement configuration. At room temperature, no signal was detected (black squares). At 50 K, a typical signal was

observed which is attributed to the inverse spin Rashba effect (ISRE). The insets in **a** and **b** show the measurement configurations. The results are measured in samples with t_{Cu} = 40 nm, t_{Au} = 5 nm, d_{SRE} = 300 nm and FM = $Co_{25}Fe_{75}$ (20 nm thick). **c,** The summarized data of control experiments. The signals of d_{SRE} = 300 nm sample measured at 50 K are also shown in **c**. The error bars indicate the standard derivation of R (R_{DSRE} and R_{ISRE}) after the magnetization is saturated. All results are measured in samples with t_{Cu} = 40 nm, t_{Au} = 5 nm, and FM = $Co_{25}Fe_{75}$ (20 nm thick). Note that the signs of SRE signals in Au/Cu samples (in which $2\Delta R_{DSRE}<0, 2\Delta R_{ISRE}>0$) are opposite to that of OEE signals in $Al_2O_3/CuO_x/Cu$ samples (in which $2\Delta R_{DOEE}>0, 2\Delta R_{IOEE}<0$) suggesting that the OEE is not the domination in the Au/Cu control devices.

Section 11. The resistivity of Cu and $Co_{25}Fe_{75}$ and the temperature dependence of bypass current

The resistivities of Cu and $Co_{25}Fe_{75}$ at various temperatures were measured using four-terminal methods. The Cu is 100 nm wide and 40 nm thick. The $Co_{25}Fe_{75}$ is 100 nm wide and 20 nm thick. The results are shown in Fig. S18.

Fig. S18

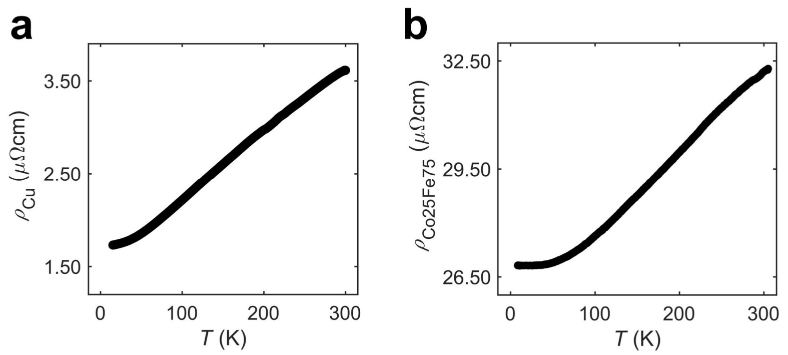

Fig. S18 | Temperature dependence of resistivity of Cu and $Co_{25}Fe_{75}$. a, b, The resistivity of Cu (**a**, ρ_{Cu}) and $Co_{25}Fe_{75}$ (**b**, ρ_{Co25Fe75}) at various temperature measured using four probe measurements.

As discussed in Supplementary Section 6, the diffusion length of the bypass current in our device is approximately 47 nm at room temperature, while the decay length of orbital accumulation exceeds 100 nm. To conclusively exclude the influence of bypass current on our estimation of the orbital accumulation decay length, we performed low-temperature COMSOL simulations to assess the temperature dependence of the bypass current. Similarly, we simulated the surface averaged value of j_{by}^{y} across the cross section of the Cu_x nanowire above the FM nanowires at various distances. The experimentally obtained temperature-dependent conductivities of Cu (Fig. S18a) and FM (Fig. S18b) were used as inputs to simulate the bypass

current at various temperatures. The simulation result at 50 K is shown in Fig. S19a suggesting an unchanged value of j_{by}^{y} at various temperature, and the diffusion length of bypass current λ_{by} as a function of temperature are shown in Fig. S19b. λ_{by} shows no temperature dependence, which is consistent with previous research[4,5], while λ_{o} decreases at lower temperatures, suggesting that the observed temperature dependent decay behavior is primarily attributed to the orbital response and is not significantly influenced by the presence of bypass current. This is because the resistivity of FM is always one order larger than that of Cu, and the bypass current can be regarded as only flowing in the Cu layer. In a single material, as the resistivity is homogenous, the bypass current distribution is determined merely by the geometric of the device[4,5].

Fig. S19

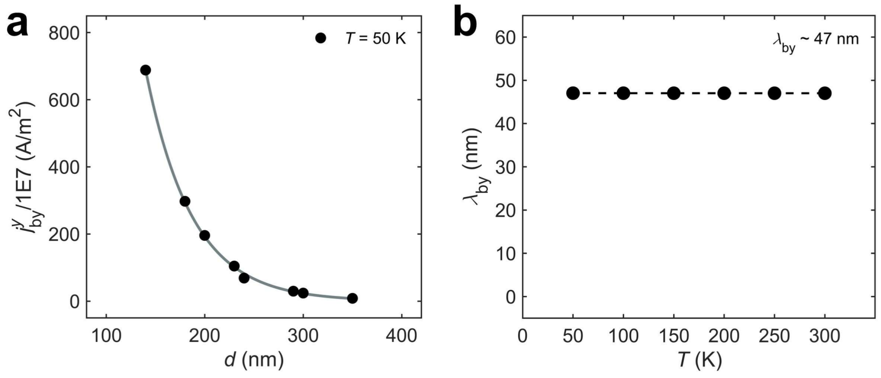

Fig. S19 | Temperature dependence of bypass current. a, The bypass current as a function of distance under 50 K. Fitting the data to exponent decay equation (grey solid curve) gives a diffusion length of bypass current λ_{by} about 47 nm, which is the same as the λ_{by} at 300 K. **b,** The temperature dependence of the diffusion length bypass current. λ_{by} remains 47 nm at various temperatures. The dash line indicates the position of $\lambda_{\mathrm{by}} = 47$ nm.

Furthermore, based on the measured resistivity we calculated the mean free path of electrons in our devices. The mean free path of electron (l_e) in Cu can be calculated as follows:

$$l_e = v_F \cdot \tau, \qquad (\text{S11}-1)$$

where v_F is the electron velocity at the Fermi surface, τ is the relaxation time.

According to Drude model and Ohm's law, τ can be calculated as follows:

$$\tau = \frac{m_e}{\rho n e^2}, \qquad (\text{S11}-2)$$

where m_e is the mass of electron, ρ is the resistivity of Cu, n is the free electron density of Cu, and e is the electron charge. Taking the parameters[13] that $n = 8.49 \times 10^{28}\ \mathrm{m}^{-3}$ and $v_F = 1.57 \times 10^{8}\ \mathrm{cm/s}$, we obtained that $l_e(T = 300\ \mathrm{K}) = 19$ nm and $l_e(T = 50\mathrm{K}) = 38$ nm.

Section 12. Temperature-dependent multiple-step hopping

We consider the hopping between the states in the oxidized Cu, which can be mediated by the metallic Cu, as illustrated in Fig. S19a. We assume that for each hopping between two neighboring grains, the probability for an electron to maintain its OAM information is

$$P = \mathrm{C}\exp(-\Delta E/k_B T) = \exp(-\alpha - \Delta E/k_B T), \qquad (\mathrm{S12}-1)$$

where $\alpha = -\ln \mathrm{C}$ is a parameter related to the wavefunction overlapping, k_B is the Boltzmann constant, and ΔE is the level mismatch between the two neighboring grains. This ΔE is related to the inelastic processes that suppress the OAM. The OAM-maintaining hopping probability across N grains is

$$P(N) = \exp(-N\alpha - N\Delta E/k_B T), \qquad (\mathrm{S12}-2)$$

and the total hopping distance is $d = Nr$, where r is the grain size. Thus, we can rewrite the hopping probability as

$$P(N) = \exp[-\alpha d/r - d\Delta E/(rk_B T)] = \exp(-d/\lambda_H), \qquad (\mathrm{S12}-3)$$

where

$$\lambda_H = \frac{r}{\alpha + \Delta E/k_B T}. \qquad (\mathrm{S12}-4)$$

Taking $\Delta E/k_\mathrm{B} = 10$ K, $r = 10$ nm, $\alpha = 1/15$, one can obtain a temperature dependence of λ_H (Fig. S19b), which is comparable with the experimental λ_o as shown in Fig. 5b in main text.

Fig. S20

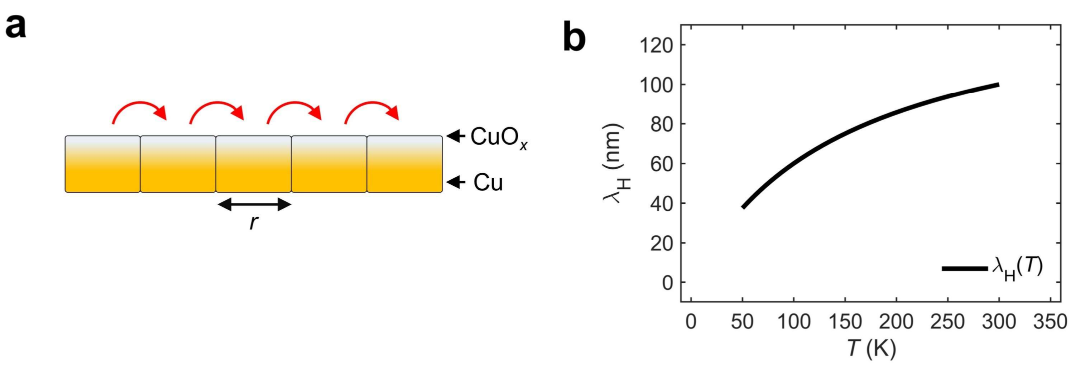

Fig. S20 | a, Schematic of the multiple hopping in Cu/CuO_x bilayer. **b,** Temperature dependence of λ_H.

Such a hopping language description corresponds to that when conductive bands involving both Cu and oxidized Cu are formed continuously across distance λ_H, the eigenstates with OAM can be created and the OAM propagation is allowed across a similar distance.

Reference

1. Kim, J. *et al.* Oxide layer dependent orbital torque efficiency in ferromagnet/Cu/oxide heterostructures. *Phys. Rev. Mater.* **7**, L111401 (2023).

2. Ding, S. *et al.* Observation of the orbital rashba-edelstein magnetoresistance. *Phys. Rev. Lett.* **128**, 67201 (2022).

3. Ding, S., Noël, P., Krishnaswamy, G. K. & Gambardella, P. Unidirectional orbital magnetoresistance in light-metal–ferromagnet bilayers. *Phys. Rev. Res.* **4**, L032041 (2022).

4. Chen, C., Tian, D., Zhou, H., Hou, D. & Jin, X. Generation and detection of pure spin current in an H -shaped structure of a single metal. *Phys. Rev. Lett.* **122**, 16804 (2019).

5. Mihajlović, G., Pearson, J. E., Garcia, M. A., Bader, S. D. & Hoffmann, A. Negative nonlocal resistance in mesoscopic gold hall bars: absence of the giant spin hall effect. *Phys. Rev. Lett.* **103**, 166601 (2009).

6. Alderson, J. E. A., Farrell, T. & Hurd, C. M. Hall coefficients of cu, ag, and au in the range 4.2-300°K. *Phys. Rev.* **174**, 729–736 (1968).

7. Pham, V. T. *et al.* Ferromagnetic/nonmagnetic nanostructures for the electrical measurement of the spin hall effect. *Nano Lett.* **16**, 6755–6760 (2016).

8. Zhang, S. & Fert, A. Conversion between spin and charge currents with topological insulators. *Phys. Rev. B* **94**, 184423 (2016).

9. Kondou, K. *et al.* Fermi-level-dependent charge-to-spin current conversion by dirac surface states of topological insulators. *Nat. Phys.* **12**, 1027–1031 (2016).

10. Isshiki, H., Muduli, P., Kim, J., Kondou, K. & Otani, Y. Phenomenological model for the direct and inverse Edelstein effects. *Phys. Rev. B* **102**, 184411 (2020).

11. Chen, X. *et al.* Giant antidamping orbital torque originating from the orbital rashba-edelstein effect in ferromagnetic heterostructures. *Nat. Commun.* **9**, 2569 (2018).

12. Pham, V. T. *et al.* Large spin-charge interconversion induced by interfacial spin-orbit coupling in a highly conducting all-metallic system. *Phys. Rev. B* **104**, 184410 (2021).

13. Kittel, C. *Introduction to Solid State Physics*. (Wiley, 2005).
